# Oncopig Soft-Tissue Sarcomas Recapitulate Key Transcriptional Features of Human Sarcomas

**DOI:** 10.1038/s41598-017-02912-9

**Published:** 2017-06-01

**Authors:** Kyle M. Schachtschneider, Yingkai Liu, Suvi Mäkeläinen, Ole Madsen, Laurie A. Rund, Martien A. M. Groenen, Lawrence B. Schook

**Affiliations:** 1 Department of Animal Sciences, University of Illinois, Urbana, Illinois USA; 2 Animal Breeding and Genomics Centre, Wageningen University, Wageningen, The Netherlands; 3 Department of Radiology, University of Illinois, Chicago, USA; 4 Department of Animal Genetics and Breeding, Sichuan Agricultural University, Chengdu, China; 5 Department of Animal Breeding and Genetics, Swedish University of Agricultural Sciences, Uppsala, Sweden

## Abstract

Human soft-tissue sarcomas (STS) are rare mesenchymal tumors with a 5-year survival rate of 50%, highlighting the need for further STS research. Research has been hampered by limited human sarcoma cell line availability and the large number of STS subtypes, making development of STS cell lines and animal models representative of the diverse human STS subtypes critical. Pigs represent ideal human disease models due to their similar size, anatomy, metabolism, and genetics compared to humans. The Oncopig encodes inducible *KRAS* ^*G12D*^ and *TP53* ^*R167H*^ transgenes, allowing for STS modeling in a spatial and temporal manner. This study utilized Oncopig STS cell line (fibroblast) and tumor (leiomyosarcoma) RNA-seq data to compare Oncopig and human STS expression profiles. Altered expression of 3,360 and 7,652 genes was identified in Oncopig STS cell lines and leiomyosarcomas, respectively. Transcriptional hallmarks of human STS were observed in Oncopig STS, including altered TP53 signaling, Wnt signaling activation, and evidence of epigenetic reprogramming. Furthermore, master regulators of Oncopig STS expression were identified, including *FOSL1*, which was previously identified as a potential human STS therapeutic target. These results demonstrate the Oncopig STS model’s ability to mimic human STS transcriptional profiles, providing a valuable resource for sarcoma research and cell line development.

## Introduction

Soft-tissue sarcomas (STS) are aggressive, often lethal mesenchymal tumors that represent roughly 1% of adult cancers and comprise approximately 50 subtypes^1^. STS arises from a number of tissue types, including muscle, fat, nerves, and blood vessels. As they are relatively uncommon, STS represent an understudied cancer type and the underlying molecular mechanisms leading to tumor formation are poorly understood. Consequently, a low 5-year survival rate of 50% is currently observed^2^, a rate that has remained unchanged for decades, highlighting the need for further research into this rare cancer type. Although a number of gene fusions are implicated in STS formation, few highly recurrent driver genes have been identified to date^3^. This has resulted in a lack of disease-specific treatments for sarcoma patients, with therapies primarily limited to surgery, radio-, and chemotherapy^4^, with additional targeted and combinational trials currently underway^2^. Although many STS patients would benefit from further research into STS diagnosis and treatment, limited STS sample availability is one of the main factors limiting their inclusion in large scale genomic studies^3^. This represents a significant challenge for STS research, as STS classifications are constantly evolving, and diagnosis of many subtypes requires molecular tests and cooperation between geneticists and pathologists^2^. Therefore, there is a need to develop STS models to deepen our understanding of sarcoma formation, detection, progression, and prognosis.

Human tumor-derived cell lines play an important role in the discovery and development of new cancer therapies^5^. These cell lines must be representative of the vast genomic variability observed in the human sarcoma population, as this variability is responsible for observed differences in treatment responses. Unfortunately, the limited availability of human sarcoma cell lines, as well as the complex genomic structures associated with the large number of STS subtypes, including chromosomal translocations and complex unstable karyotypes, has hampered sarcoma research^6^. Therefore, there is a need to develop model STS cell lines representative of the diverse human sarcoma subtypes. Production of such cell lines represents a crucial step in understanding the mechanisms behind the variable responses to targeted therapies observed across STS subtypes^3, 6^.

In addition to cell lines, *in vivo* models are also needed to translate *in vitro* treatment developments and discoveries to clinical settings. While xenografts produced by injecting human STS cell lines into immunocompromised mice are frequently used to model human sarcomas, potential treatments identified in these model systems have not translated to clinical settings^3^. This is due to a number of factors, including introduction of new mutations in culture, difficulties in modeling interactions between tumor cells and host microenvironment, and the inability to study early tumor formation events. Alterative genetically engineered mouse models that reproduce characteristics of human STS are also commonly used in sarcoma research. These include transgenic murine models containing *Pten* ^7^ or *Kras* and *Trp53* ^8^ mutations that can develop a number of STS subtypes. While genetically engineered small animal models may be useful for a variety of studies including biomarker discovery and therapeutic testing, animal models that more closely mimic humans in terms of anatomy, physiology, genetics, and epigenetics are needed to address a range of unmet clinical cancer needs, including improving diagnostic imaging and locoregional combination therapies.

Pigs represent an ideal large animal models for human diseases due to their similar size, anatomy, metabolism, genetics and epigenetics compared to humans^9, 10^. Pigs are less expensive and ethically more acceptable than canines and non-human primates, and porcine studies are more predictive of therapeutic treatments in humans than rodents^11^. The recent production of a transgenic porcine cancer model, referred to as the Oncopig^12^, provides the opportunity to study biomarker discovery, treatment development/response, functional genomics, and provide surgical training in a model highly similar to humans. The Oncopig model encodes Cre recombinase inducible porcine transgenes encoding *KRAS* ^*G12D*^ and *TP53* ^*R167H*^, which represent a commonly mutated oncogene and tumor suppressor in human cancers, respectively^12^. This allows the Oncopig to model a wide variety of human sarcomas in an inducible, temporal manner. Although *KRAS* is rarely mutated in human STS^3^, *TP53* is among the most frequently mutated genes identified^13, 14^, and deregulation of both *TP53* and *KRAS* signaling pathways is commonly utilized to produce sarcoma animal models^8, 14^. This strategy has been used to develop a porcine model that produces spontaneous osteosarcomas due to constitutive *TP53* ^*R167H*^ expression^15^. Unlike this model, the Oncopig allows for spatial and temporal induction of STS cell lines and tumors originating from a wide variety of cell types, helping fill the gap created by the lack of relevant human sarcoma-derived cell lines and tissue samples available.

This study validates the Oncopig as a suitable model for human STS studies through the identification of similar alterations in transcriptional regulation in Oncopig and human STS. Towards this end, commonly identified alterations in gene expression and pathway regulation in human STS, including altered TP53 signaling, activation of Wnt signaling, and epigenetic reprogramming were investigated in Oncopig STS cell lines (fibroblast) and tumors (leiomyosarcoma). The results presented here demonstrate the validity of the Oncopig model to mimic human STS on the transcriptomic level, providing further validation for the use of the Oncopig in sarcoma research.

## Results and Discussion

### Study Design

This study sought to validate the Oncopig STS model as a suitable model for preclinical STS research by identifying similar alterations in transcriptional regulation in Oncopig and human STS. Transformed Oncopig fibroblast cell lines from a previously published study were utilized to profile Oncopig STS gene expression *in vitro* ^12^. The primary fibroblasts isolated from four Oncopigs stained positively with vimentin and negatively for cytokeratin, confirming their mesenchymal origin (Supplementary Fig. S1). Briefly, primary fibroblast cell lines were treated with adenoviral particle vectors encoding Cre recombinase (AdCre), resulting in the transformation of cells in culture^12^. The transformed fibroblasts (referred to as STS cell lines) were characterized by loss of contact inhibition, increased cell migration, and reduced cell cycle length compared to primary fibroblasts. In contrast to the spindle shaped, tightly adherent primary fibroblasts, STS cells were smaller, rounder, and less adherent. These phenotypic changes are characteristic of cellular dedifferentiation and transformation, which, in addition to tumor formation following injection in immunocompromised mice, support their tumorigenic nature^12^.

In addition, previously produced RNA-seq data from three Oncopig skeletal muscle and four STS tumor samples were utilized to profile Oncopig STS gene expression *in vivo* ^12^. Briefly, Oncopig STS tumors were produced via intramuscular injection of AdCre into the Oncopig hind legs, resulting in the formation of tumor masses visible by ultrasound within 10 days. Their mesenchymal origin was confirmed through blinded pathological analysis and positive vimentin staining, resulting in the classification of the tumors as leiomyosarcomas. The Oncopig leiomyosarcomas contained densely cellular, non-encapsulated, and locally infiltrative neoplasm, and cells were arranged in bundles supported by a fibrous stroma. The neoplastic tumor cells were pleomorphic with single to multiple nuclei, with areas of necrosis and inflammation identified throughout the tumors. Together, these samples were used to identify altered transcriptional regulation in the Oncopig STS model both *in vitro* and *in vivo*.

### Cre recombinase induces mutant transgene expression

**Figure 1:**
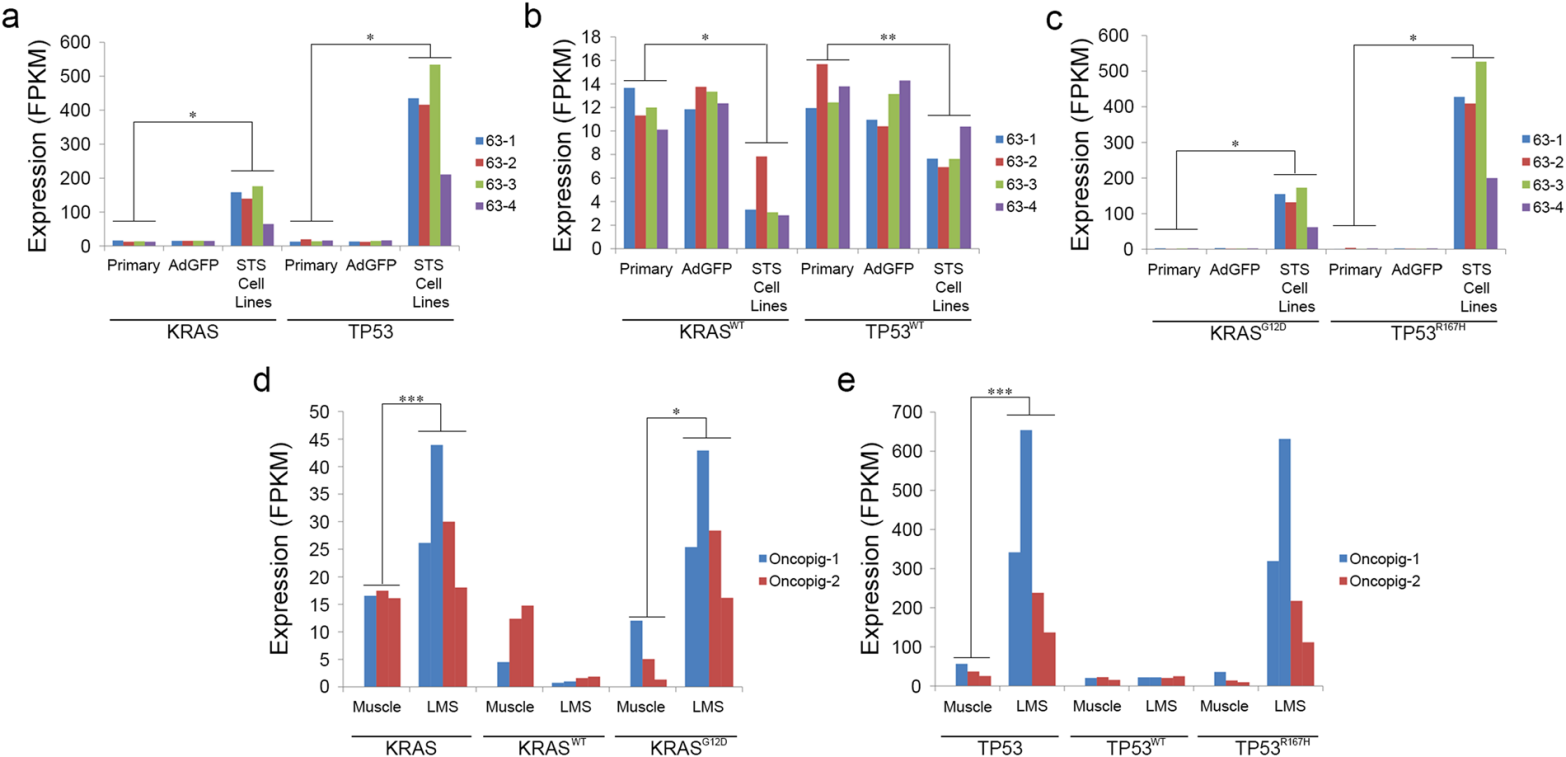
Expression of *KRAS* and *TP53* across Oncopig STS cell lines and leiomyosarcoma tumors. Expression of (**a**) total, (**b**) wild type (WT), and (**c**) mutant *KRAS* and *TP53* in Oncopig primary fibroblasts, AdGFP treated fibroblasts, and STS cell lines. Expression of total, WT, and mutant (**d**) *KRAS* and (**e**) *TP53* in Oncopig skeletal muscle and leiomyosarcomas (LMS). Samples represent biological replicates. Expression values are presented as fragments per kilobase of transcript per million fragments mapped (FPKM). *denotes p < 0.05, **denotes p < 0.01, ***denotes q < 0.05.

Mutant transgene expression in the Oncopig STS model was evaluated via RNA-seq. While exposure to adenoviral particle vectors encoding GFP (AdGFP) had no effect on total, wild type (WT), or mutant *KRAS* ^*G12D*^ and *TP53* ^*R167H*^ expression *in vitro* (Fig. 1a,b,c), increased total *KRAS* and *TP53* expression was observed in the AdCre treated STS cell lines compared to primary fibroblasts (p = 0.016 and 0.011, respectively; Fig. 1a). In addition, significantly higher mutant *KRAS* ^*G12D*^ and *TP53* ^*R167H*^ expression (p = 0.013 and 0.011, respectively), and significantly lower WT *KRAS* and *TP53* expression (p = 0.029 and 0.003, respectively) were observed in the STS cell lines compared to primary fibroblasts (Fig. 1b,c). Increased expression of both *KRAS* and *TP53* was also observed in the Oncopig leiomyosarcomas compared to *in vivo* controls (0.82 and 3.10 log2 fold change, respectively; Fig. 1d,e). Additionally, although reduced WT *KRAS* expression was observed in the leiomyosarcomas, the results were not significant (p = 0.095; Fig. 1d). However, significantly higher expression of mutant *KRAS* ^*G12D*^ was observed in the leiomyosarcomas (p = 0.026; Fig. 1d). Increased mutant *TP53* ^*R167H*^ expression was also observed in the leiomyosarcomas, although the result was not significant due to the high level of variation in mutant *TP53* ^*R167H*^ expression observed amongst the samples (p = 0.075; Fig. 1e). No difference in WT *TP53* expression was observed between the leiomyosarcomas and controls. Together, these results demonstrate induction of mutant *KRAS* ^*G12D*^ and *TP53* ^*R167H*^ expression in Oncopig cells following exposure to Cre recombinase.

### Reproducible effect and temporal stability of transgene expression on gene expression profiles

**Figure 2:**
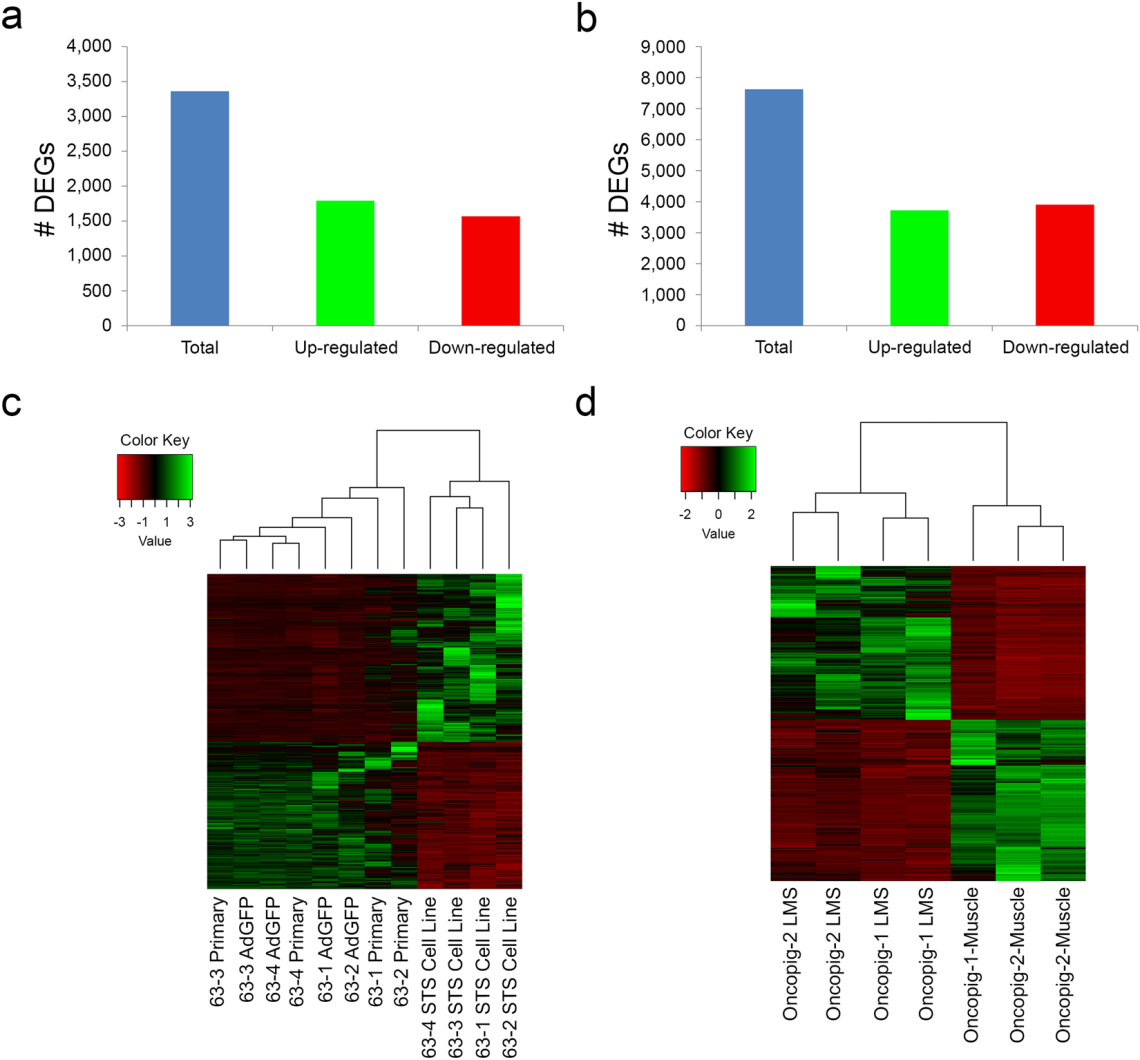
Total number of DEGs and their expression levels across biological replicates. Total number of DEGs, including those displaying elevated and reduced expression in (**a**) Oncopig STS cell lines compared to controls and (**b**) leiomyosarcoma tumors compared to controls. (**c**) Heatmap of the normalized expression level of the 3,360 DEGs for each cell line, represented as z-scores. (**d**) Heatmap of the normalized expression level of the 7,625 DEGs for each *in vivo* sample, represented as z-scores. LMS = leiomyosarcoma. Dendrograms represent relationships between samples based on complete linkage clustering.

As incubating Oncopig primary fibroblast cell lines with AdGFP had no effect on *KRAS* or *TP53* expression, both primary and AdGFP treated fibroblasts were used as controls in comparisons with STS cell lines. A total of 3,360 differentially expressed genes (DEGs) were detected between the STS and control cell lines, with 1,792 and 1,568 DEGs displaying elevated and reduced expression in the STS lines, respectively (Fig. 2a, Supplementary Table S1). In addition, a total of 7,625 DEGs were identified between the leiomyosarcomas and control samples, with 3,721 and 3,904 displaying elevated and reduced expression in the leiomyosarcomas, respectively (Fig. 2b, Supplementary Table S2). Cluster analysis based on the relative DEG expression levels resulted in both *in vitro* and *in vivo* samples clustering by group (Fig. 2c,d), demonstrating the reproducible effect of mutant *KRAS* ^*G12D*^ and *TP53* ^*R167H*^ expression on Oncopig STS expression profiles. In addition, the expression profile for one STS line (12 passages) was compared to the expression profile of the same line after culturing for 99 passages to investigate the temporal stability of Oncopig STS expression profiles *in vitro*. The expression profiles of the two time points were highly correlated when comparing the 22,499 genes with expression information for both time points (Spearman’s Rho 0.92, p < 1 × 10^−15^; Supplementary Fig. S2), demonstrating the stability of the Oncopig STS cell line over time. The combination of reproducibility and temporal stability highlights the ability to produce stable Oncopig cell lines critically needed for *in vitro* STS research. Currently characterized human STS subtypes do not include transformed fibroblasts such as the Oncopig STS cell lines discussed here. Thus the results provided here demonstrate that reproducible stable transformation of Oncopig mesenchymal cells will allow for further production of other Oncopig STS cell lines representative of human STS subtypes from additional cell lineages.

### Altered *TP53* signaling

**Figure 3:**
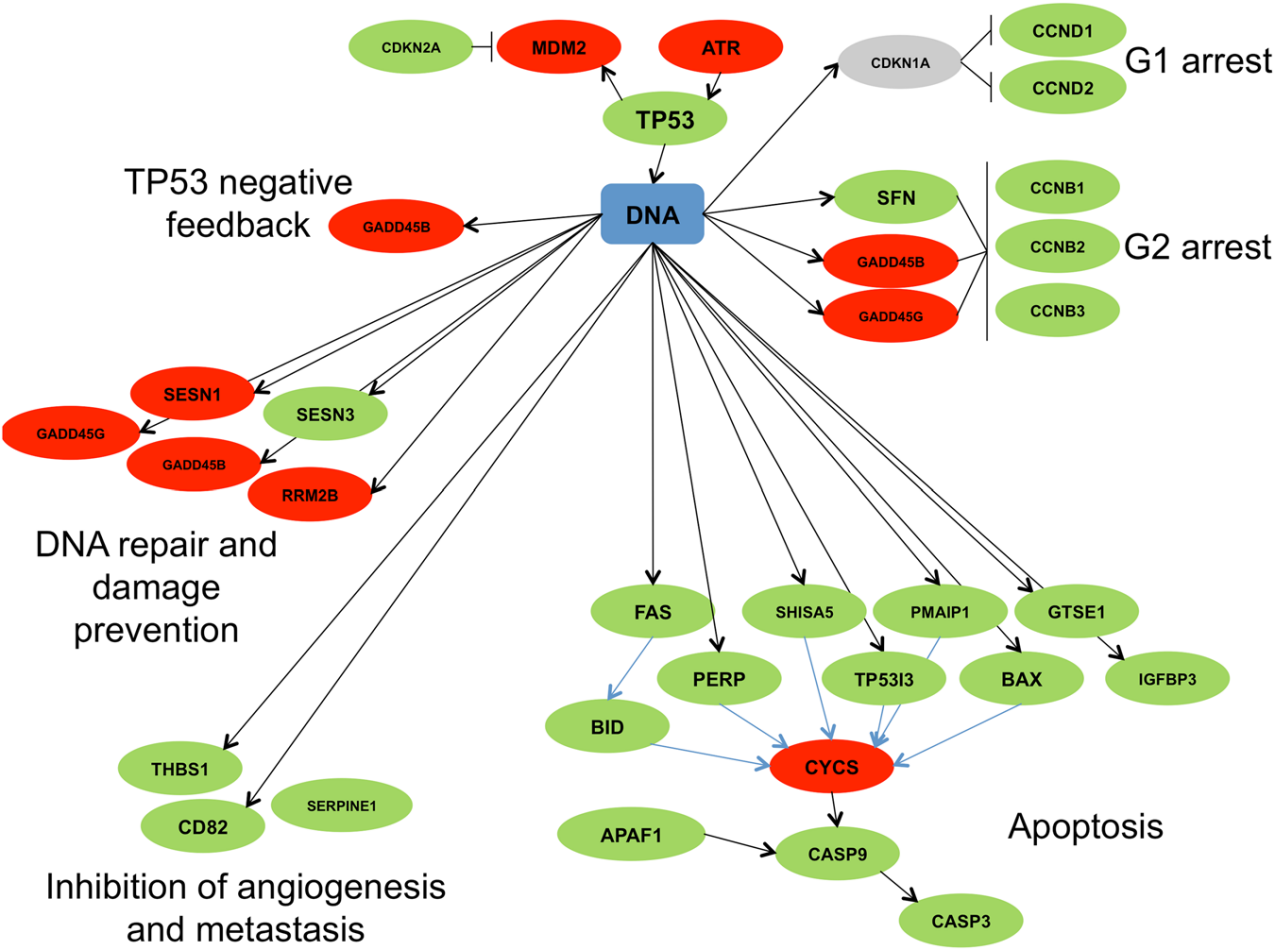
Map of DEGs in Oncopig leiomyosarcomas and their functions within the *TP53* signaling pathway. Adopted from the KEGG hsa04115 p53 signaling pathway. Green ovals represent genes with elevated expression, red ovals represent genes with reduced expression, and grey ovals represent genes with no expression change in Oncopig leiomyosarcomas compared to controls. Black bars represent inhibition, black arrows represent activation, and blue arrows represent indirect effects.

**Figure 4:**
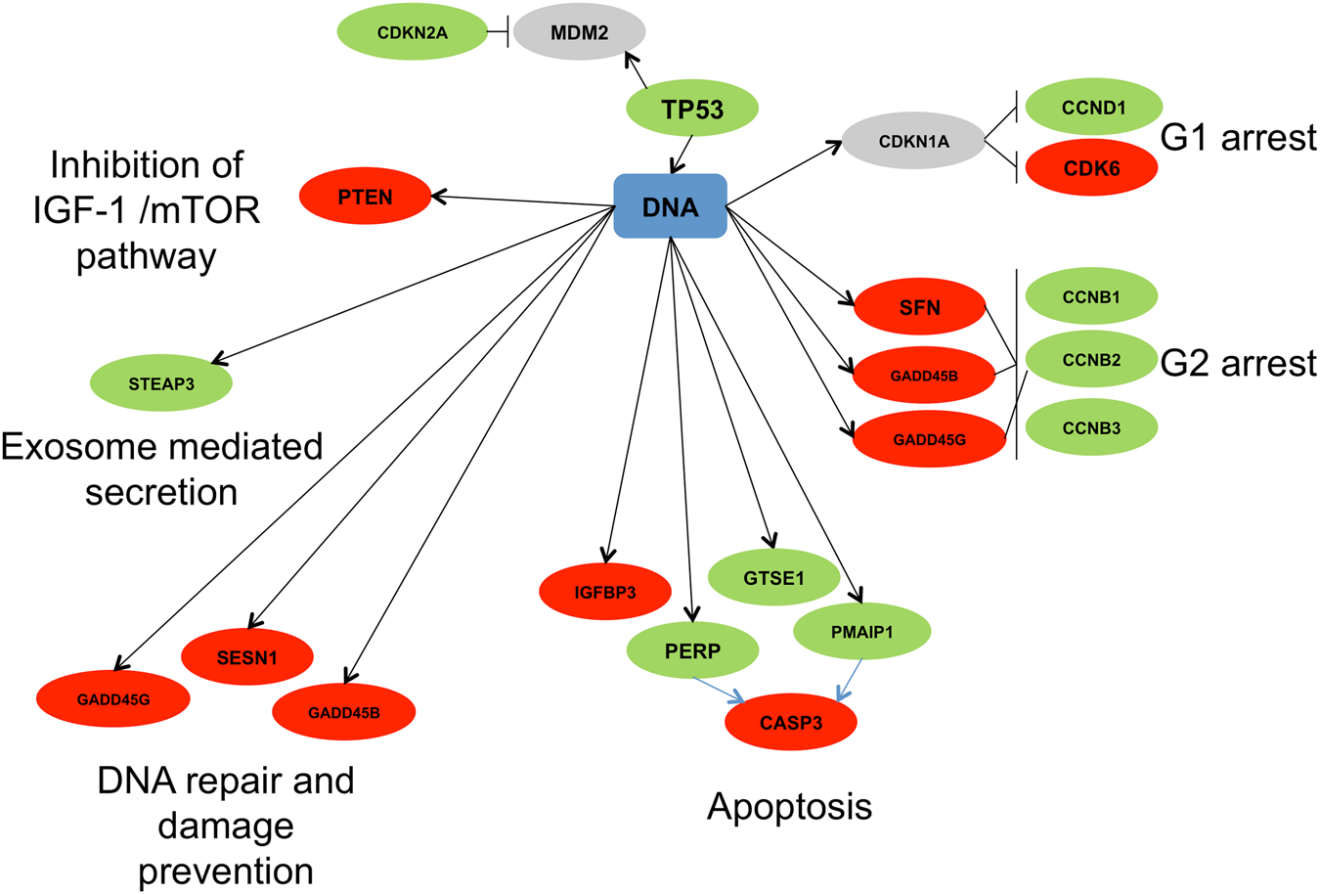
Map of DEGs in Oncopig STS cell lines and their functions within the *TP53* signaling pathway. Adopted from the KEGG hsa04115 p53 signaling pathway. Green ovals represent genes with elevated expression, red ovals represent genes with reduced expression, and grey ovals represent genes with no expression change in Oncopig STS cell lines compared to controls. Black bars represent inhibition, black arrows represent activation, and blue arrows represent indirect effects.

*TP53* is among the most frequently mutated genes identified in human STS^13^ including leiomyosarcomas^16, 17^, and disruption of the *TP53* pathway is a well characterized event in human STS^18^. Disruptions in *TP53* signaling result in uncontrolled proliferation and escape from cell death through altered regulation of the cell cycle and apoptosis, which represent crucial steps in tumor development and progression. Therefore, it was hypothesized that altered *TP53* signaling would be observed if the transcriptional profile of the Oncopig STS model truly reflects human STS. As expected, the *TP53* signaling pathway was one of the KEGG pathways enriched for genes with elevated expression levels in the Oncopig leiomyosarcomas (Fig. 3, Supplementary Table S3), with a total of 24 and 7 genes displaying elevated and reduced expression in the pathway, respectively. In addition, 18 genes within this pathway were differentially expressed in the Oncopig STS cell lines (10 elevated, 8 reduced, Fig. 4, Supplementary Table S1), although the pathway was not significantly enriched.

Reduced cell cycle length was previously reported in the Oncopig STS cell lines^12^, and is commonly observed due to disturbances in the *TP53* pathway in human STS including leiomyosarcomas^19^. This occurs through *TP53* dependent regulation of cyclin D and B, whose expression contributes to loss of cell cycle regulation by promoting the G1-to-S and G2-to-M transitions, respectively^20, 21^. Consistent with humans, increased cyclin B (*CCNB1*, *CCNB2*, and *CCNB3*) and D (*CCND1*) expression was observed in both Oncopig leiomyosarcomas (log2 fold change 1.45, 1.37, 1.65, and 2.1, respectively) and STS cell lines (log2 fold change 1.11, 1.06, 1.07, and 1.17, respectively). In addition, increased *CCND2* expression (log2 fold change 2.14) was observed in the Oncopig leiomyosarcomas. The increased cyclin expression coincided with reduced expression of the cyclin B inhibitors *GADD45B* and *GADD45G* in both Oncopig leiomyosarcomas (log2 fold change −1.12 and −2.03, respectively) and STS cell lines (log2 fold change −2.42 and −2.8, respectively), in addition to the cyclin B inhibitor *SFN* (log2 fold change −2.36) in the STS cells lines. Together, these results provide a mechanism for the previously observed reduced cell cycle length in Oncopig STS cell lines^12^, and highlight fundamental similarities in cell cycle deregulation between human and Oncopig STS. In addition, *GADD45B* and *GADD45G* are also important for *TP53* dependent DNA repair and damage prevention^22^. Thus, reduced expression of these genes, in addition to *SESN1* in both Oncopig STS cell lines and leiomyosarcomas (log2 fold change −1.26 and −1.83, respectively) also suggests reduced *TP53* dependent DNA repair and damage prevention in Oncopig STS. Therefore, investigation into the accumulation of somatic mutations and chromosomal instability may be of interest for future Oncopig STS studies.

Another important role of *TP53* signaling is the induction of apoptosis, with evasion of apoptosis via altered *TP53* signaling commonly observed in human STS^23^. This occurs through increased expression of *GTSE1 in leiomyosarcomas* ^21^, a negative regulator of *TP53* involved in the repression of *TP53* induced apoptosis, as well as reduced expression of the proapoptotic factors *IGFBP3* ^24^ and *CASP3* in osteosarcomas^25^. Consistent with observations in human STS, *GTSE1* expression was elevated in the Oncopig leiomyosarcomas and STS cell lines (log2 fold change 0.93 and 0.83, respectively), in addition to reduced *IGFBP3* and *CASP3* expression in the Oncopig STS cell lines (log2 fold change −4.63 and −0.64, respectively), suggesting evasion of *TP53* dependent apoptosis in Oncopig STS. However, although reduced apoptotic activity is associated with cancer progression, apoptotic cells are still present in tumors with up to 15% reported to undergo apoptosis in human STS^26^. This addresses why expression of some *TP53* dependent proapoptotic factors including *BAX* are commonly observed in human leiomyosarcomas^27^. Indeed, increased *BAX* expression was observed in the Oncopig leiomyosarcomas (log2 fold change 1.93), in addition to a number of other *TP53* dependent proapoptotic factors, including *PMAIP1* (log2 fold change 1.59), *PERP* (log2 fold change 1.76), *BID* (log2 fold change 1.37), *TP53I3* (log2 fold change 2.69), *SHISA5* (log2 fold change 0.91), *IGFBP3* (log2 fold change 4.26), *APAF1* (log2 fold change 1.11)*, CASP9* (log2 fold change 0.95), and *CASP3* (log2 fold change 1.83). The expression of these proapoptotic factors in the Oncopig leiomyosarcomas suggests high levels of apoptotic cells were present in the samples collected for sequencing. This theory is supported by the pathology reports identifying areas of necrosis and chronic inflammation throughout the tumors^12^ and also explain why elevated expression of these factors was not observed in the Oncopig STS cell lines. Together, these results demonstrate similar deregulation of *TP53* dependent signaling between Oncopig and human STS suggestive of altered cell cycle and apoptotic regulation.

### Activation of Wnt signaling

**Figure 5:**
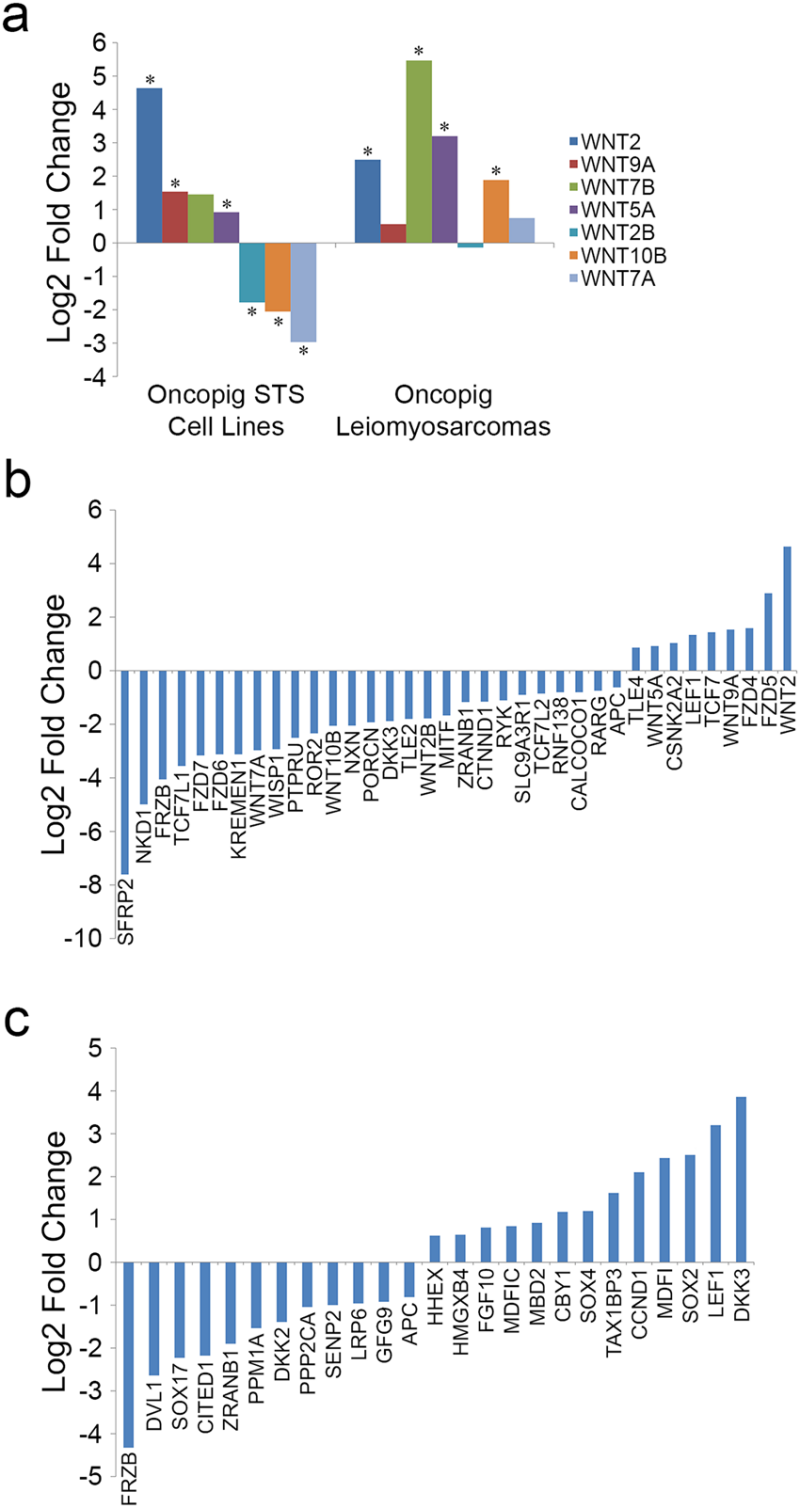
Differential expression of genes involved in Wnt signaling in Oncopig STS. (**a**) Expression of WNTs in Oncopig leiomyosarcomas and STS cell lines relative to controls, represented as the log2 fold change. (**b**) DEGs involved in the Wnt receptor signaling pathway in the Oncopig STS cell lines, represented as the log2 fold change relative to control. (**c**) DEGs involved in the regulation of Wnt receptor signaling pathway in the Oncopig leiomyosarcomas, represented as the log2 fold change relative to control. *denotes q-value < 0.05.

Wnt signaling is critical for normal development and adult tissue homeostasis, and aberrant Wnt pathway activation is observed in over half of human STS^28^. Although human STS with upregulated Wnt signaling display a variety of Wnt expression profiles, each expresses elevated levels of at least one Wnt gene, with downregulation of Wnt signaling leading to inhibition of sarcoma cell proliferation^28^. These results suggest Wnt signaling plays an essential role in driving human STS development, and therefore altered Wnt signaling is expected to occur during Oncopig STS development. As expected, increased Wnt gene expression was observed during Oncopig STS development, with 4 and 3 Wnt genes displaying elevated expression in the Oncopig leiomyosarcomas and STS cell lines, respectively (Fig. 5a).

In addition to the altered Wnt gene expression profiles, the Wnt receptor signaling pathway was significantly enriched for DEGs in the Oncopig STS cell lines (Fig. 5b, Supplementary Table S4). This included the reduced expression of Wnt pathway inhibitors commonly downregulated in human STS, including *FRZB* (log2 fold change −4.06)^29^, *TCF7L1* (log2 fold change −3.56)^30^, *DKK3* (log2 fold change −1.88)^31^, *CTNND1* (log2 fold change −1.15)^32^, and *TCF7L2* (log2 fold change −0.85)^30^. Of particular interest is *SFRP2* (log2 fold change −7.61), a Wnt pathway inhibitor whose expression was the most severely reduced of the DEGs in the Wnt signaling pathway. This gene is epigenetically silenced in human sarcomas, resulting in activation of Wnt signaling^33^. In addition, a number of Wnt receptor signaling genes commonly expressed at high levels in human STS also displayed elevated expression in the Oncopig STS cell lines, including *TLE4* (log2 fold change 0.86)^34^, *LEF1* (log2 fold change 1.34)^35^, and *TCF7* (log2 fold change 1.44)^36^.

Although the Wnt receptor signaling pathway was not enriched for DEGs in the Oncopig leiomyosarcomas, enrichment of the regulation of Wnt receptor signaling pathway was observed (Fig. 5c, Supplementary Table S5). The observed reduced gene expression in this pathway is also characteristic of human leiomyosarcomas, including reduced expression of *FRZB* (log2 fold change −4.32)^37^, as well as *DKK2* (log2 fold change −1.39), which is epigenetically silenced in a number of human STS subtypes including leiomyosarcomas^28^. In addition, elevated expression observed in the Oncopig leiomyosarcomas is also consistent with human STS, including increased *SOX4* (log2 fold change 1.19) and *SOX2* expression (log2 fold change 2.5), which promote proliferation and invasion in human osteosarcomas^38, 39^, *LEF1* (log2 fold change 3.2), whose expression is associated with a number of human STS subtypes^35^, and the previously mentioned *CCND1* (log2 fold change 2.1). Together, the observed similarities in Wnt signaling activation between Oncopig and human STS suggests the Oncopig may be an ideal model to test novel STS therapies targeting the Wnt signaling pathway.

### Evidence of epigenetic reprogramming

The role of genome-wide epigenetic reprogramming in the development of human cancers is well established, and epigenetic regulators are emerging as promising therapeutic targets for STS^40^. One of the most well studied forms of epigenetic regulation is DNA methylation, which is maintained by DNA methyltransferases (DNMT) and altered in a wide variety of human STS including leiomyosarcomas^41^. Although DNA methylation analysis was not performed in this study, altered expression of DNMTs is linked to altered genome-wide DNA methylation patterns^42^. Therefore, the expression levels of epigenetic regulators were investigated to provide indirect evidence of epigenetic reprogramming in Oncopig STS. As expected, altered DNMT expression was observed in the Oncopig STS model, with increased *DNMT1* (log2 fold change 0.7) expression in the Oncopig STS cell lines, and increased *DNMT1* and *DNMT3L* (log2 fold change 0.73 and 2.37, respectively), as well as decreased *DNMT3A* (log2 fold change −1.35) expression in the Oncopig leiomyosarcomas. These results suggest that as in human STS, genome-wide alterations in DNA methylation patterns exist in Oncopig STS.

The lack of differential expression of the same DNA methyltransferases in the Oncopig leiomyosarcomas and STS cell lines, as well as differences in the above-mentioned pathways is likely due to a number of factors, including differential cell origin of both the *in vitro* and *in vivo* control and tumorigenic samples, resulting in modeling of different STS subtypes, as well as differences in *in vitro* and *in vivo* microenvironments. The effects of the differential *in vitro* and *in vivo* microenvironments on Oncopig STS transcriptional profiles is evidenced by the above mentioned areas of necrosis and chronic inflammation throughout the leiomyosarcoma tumors^12^ that were not present in the STS cell line cultures.

In addition to DNA methylation, histone modifications represent another crucial form of gene regulation, and therapeutics targeting histone modifying enzymes are also being investigated for STS treatments^40^. The histone methyltransferase *EZH2* methylates histone H3 lysine 27^43^, and has been identified as an oncogene in human STS, with elevated expression observed across subtypes^40^. Furthermore, knockdown of *EZH2* induces cell death and inhibits tumor growth^44^, suggesting that *EZH2* may be an effective therapeutic target for STS. Consistent with observations in human STS, elevated *EZH2* expression was observed in both the Oncopig leiomyosarcomas and STS cell lines (log2 fold change 0.89 and 0.74, respectively), suggesting the presence of altered histone modification patterns in Oncopig STS. Together these results, in addition to the above mentioned reduced expression of genes known to be epigenetically silenced in human STS, provide evidence for similar genome-wide epigenetic reprogramming in Oncopig and human STS, suggesting the Oncopig may be an ideal model to test therapies targeting epigenetic regulators. However, further epigenetic studies are required to confirm altered epigenetic regulator expression results in genome-wide epigenetic reprogramming in Oncopig STS, as well as their similarity to alterations observed in human STS subtypes.

### Master regulators of Oncopig STS expression

Master regulators of cancer are transcription factors that play key roles in regulating genes that show aberrant expression in tumors and that are implicated in cancer development. In order to identify master regulators of Oncopig STS, transcriptional regulatory networks were reverse-engineered from gene expression profiles. A number of master regulators were identified in the Oncopig STS cell lines, including *FOSL1*, which was predicted to regulate 49% of the genes with elevated expression in the Oncopig STS cell lines (Table 1). *FOSL1* is a key transcriptional regulator in human STS, with inhibitors of *FOSL1* showing promise as osteosarcoma therapies^45^. *TP53* is also believed to regulate *FOSL1* expression^46^, providing an indirect link between mutant *TP53* ^*R167H*^ expression and nearly half of the elevated gene expression identified in the Oncopig STS cell lines. In addition, *SRF*, a transcription factor that stimulates cell differentiation was predicted to regulate 40% of genes with reduced expression in the Oncopig STS cell lines (Table 1). Although *SRF* has not been implicated in human STS development, these results suggest reduced *SRF* expression is responsible for a significant proportion of the observed reduced gene expression, and may be worth further investigation in human STS.

**Table 1:** Master regulators of Oncopig STS cell lines.

Transcription Factor	NES	# DE Target Genes
**Master Regulators of Elevated Gene Expression**		
FOSL1	6.603	873
**Master Regulators of Reduced Gene Expression**		
SRF	5.721	628
ABCF2	4.015	71

Transcription factors whose target genes were enriched (normalized enrichment score (NES) > 4) among the differentially expressed (DE) genes displaying either elevated or reduced expression.

Master regulators were also identified in the Oncopig leiomyosarcomas, including *MEF2C*, which was predicted to regulate 48% of genes with reduced expression in the Oncopig leiomyosarcomas (Table 2). This is consistent with the recurrent repression of *MEF2C* target genes and the hypothesis that *MEF2* acts as a tumor suppressor in human leiomyosarcoma^47^. Furthermore, human STS with reduced *MEF2C* target gene expression fall into two groups, those with reduced *PTEN* expression, and those with reduced *HDAC4* expression^47^. The Oncopig leiomyosarcomas also displayed reduced *HDAC4* levels, but not reduced *PTEN* expression, suggesting this model may be suitable for studies focused on the latter STS subgroup. In addition, *SPI1* and *ETV4* were predicted to regulate 53% and 51% of genes with elevated expression in the Oncopig leiomyosarcomas, respectively (Table 2). *SPI1* promotes differentiation blockage and malignant transformation by inhibiting *TP53* activity^48^, and although not previously implicated in human leiomyosarcoma development, is expressed at elevated levels in human dendritic cell sarcomas^49^. In addition, increased *ETV4* expression, as well as gene fusions between *ETV4* and *EWSR1* or *EWS*, are observed in human Ewing sarcomas^50–52^, highlighting the importance of these transcription factors in human STS development. Together, the identification of transcription factors previously implicated in human STS suggests Oncopig STS transcription profiles are controlled by the same master regulators as human STS. These results also provide novel potential therapeutic targets for STS.

**Table 2:** Master regulators of Oncopig leiomyosarcomas.

Transcription Factor	NES	# DE Target Genes
**Master Regulators of Elevated Gene Expression**		
SPI1	7.128	1,977
ETV4	5.322	1,897
UBB	4.702	1,080
HMGA1	4.114	1,145
FOS	4.061	432
EXOSC3	4.022	1,050
**Master Regulators of Reduced Gene Expression**		
MEF2C	7.593	1,893
HLF	4.332	1,527

Transcription factors whose target genes were enriched (normalized enrichment score (NES) > 4) among the differentially expressed (DE) genes displaying either elevated or reduced expression.

Although a number of the master regulators identified in Oncopig leiomyosarcomas are implicated in human STS development, they have not been previously identified in human leiomyosarcomas. The same is true for a number of the gene expression changes identified in Oncopig leiomyosarcomas and STS cell lines. This may be due to differences between Oncopig and human STS, lack of annotation of genes of interest in the current pig genome assembly, or the relatively few number of studies focused on molecular profiling of human STS, including leiomyosarcomas. Therefore, further work is required to confirm the role of identified genes and master regulators in both Oncopig and human STS.

### Leiomyosarcoma transcriptional hallmarks

To further confirm recapitulation of human leiomyosarcoma transcriptional hallmarks in Oncopig leiomyosarcomas, Oncopig leiomyosarcoma expression profiles were assessed for gene expression changes previously identified in human leiomyosarcomas. Of the 18 genes that consistently displayed altered expression in a previous comparison between 11 human leiomyosarcomas and control tissues^53^, 15 were annotated in the pig genome, 10 of which displayed the same directional expression change in Oncopig leiomyosarcomas (Supplementary Table S6). In addition, altered gene expression was used to identify human leiomyosarcoma subclasses, referred to as the neural differentiation, inflammatory related, and simplification clusters^53^. While only 12 of the 48 neural differentiation genes annotated in the pig genome displayed the same expression patterns in Oncopig leiomyosarcomas as human leiomyosarcomas, 8 out of 9 inflammatory related and 7 out of 10 simplification genes displayed the same expression patterns in Oncopig leiomyosarcomas (Supplementary Table S6). In addition, a number of genes found to be overexpressed in human leiomyosarcomas^54–56^ were overexpressed in Oncopig leiomyosarcomas (Supplementary Table S6), indicating Oncopig leiomyosarcomas display similar gene expression changes as human leiomyosarcomas.

Although a number of genes displayed similar expression changes in Oncopig and human leiomyosarcomas, this was not the case for all genes profiled. This may be due to differential expression profiling techniques (RNA-seq vs arrays), sample processing and storage (frozen vs formalin fixed paraffin embedded), and the relatively few number of studies focused on molecular profiling of leiomyosarcomas, which tend to include a mixture of STS subtypes^57^. In addition, many human leiomyosarcoma gene signatures are based on comparisons with other STS subtypes or different control tissues than in this study^53–56^. Therefore, further analysis is required to confirm Oncopig leiomyosarcomas display altered gene expression observed in human leiomyosarcomas relative to other human STS subtypes.

### Independent validation of RNA-seq results

**Figure 6:**
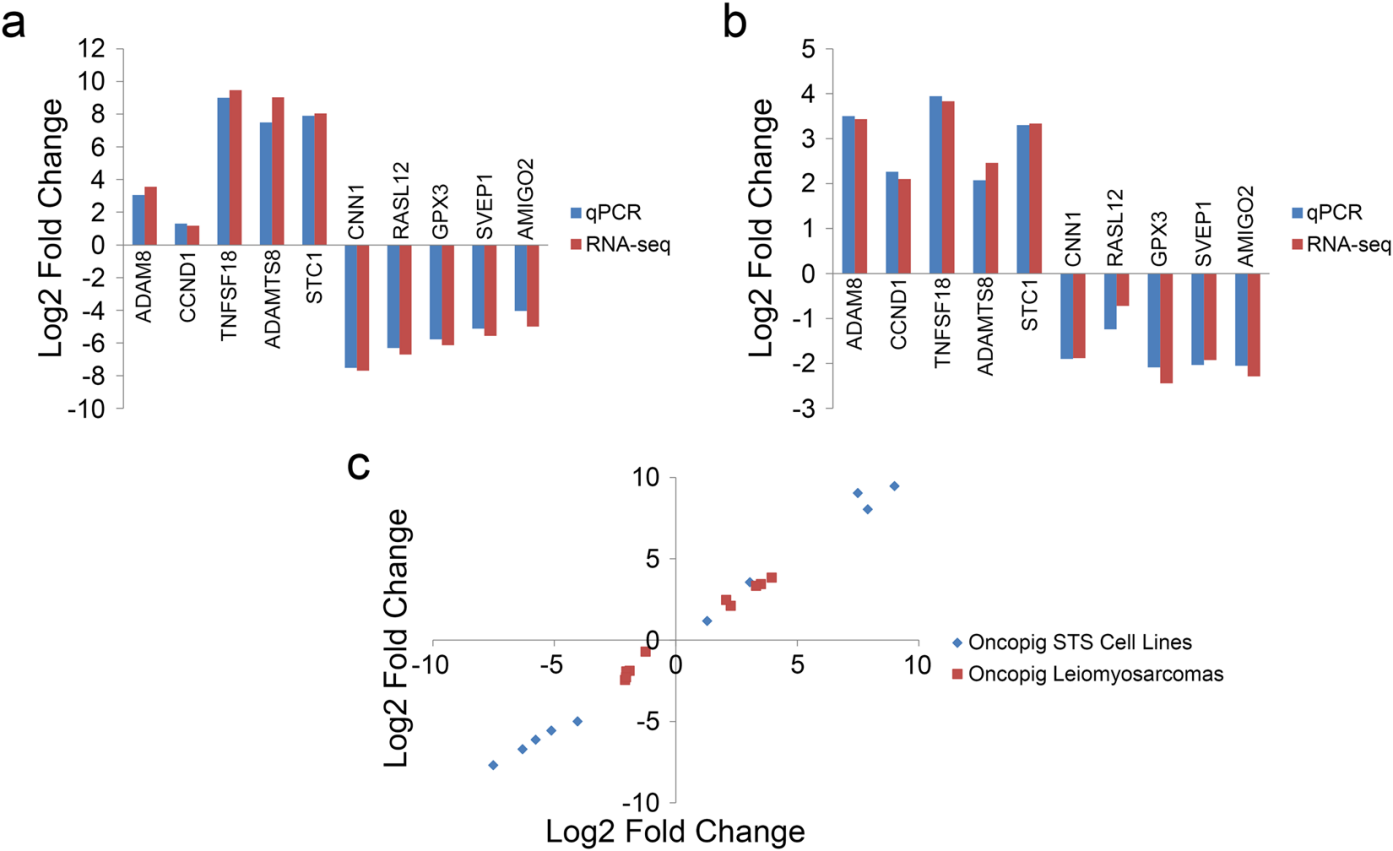
Independent validation confirms RNA-seq results. Log2 fold changes assessed using PCR and RNA-seq for 5 genes displaying elevated and reduced expression in the (**a**) Oncopig STS cell lines relative to controls and (**b**) leiomyosarcomas relative to controls. (**c**) Log2 fold changes assessed using qPCR and RNA-seq were highly correlated (Spearman’s Rho 0.992, p = 2.2 × 10^−16^).

In order to validate the reported RNA-seq gene expression results, the log2 fold changes for five up- and five down-regulated DEGs were determined using qPCR on the same set of samples. The results revealed the same directional change in expression for all genes tested in both the *in vitro* and *in vivo* sample comparisons (Fig. 6a,b). In addition, the log2 fold changes measured by RNA-seq and qPCR were highly correlated (Spearman’s Rho 0.992, p = 2.2 × 10^−16^; Fig. 6c), further validating the gene expression results presented here.

## Conclusions

The results presented here demonstrate the Oncopig model’s ability to mimic human STS on the transcriptomic level, further supporting the use of the Oncopig as a qualified alternative translational model for sarcoma research. Commonly identified alterations in gene expression and pathway regulation in human STS, including altered TP53 signaling, activation of Wnt signaling, and evidence of epigenetic reprogramming were identified in Oncopig leiomyosarcomas and STS cell lines. Furthermore, the identification of master regulators previously implicated in human STS development further demonstrates the relevance of this model, and also provides novel potential therapeutic targets. Finally, the reproducible and temporally stable effect of mutant *KRAS* ^*G12D*^ and *TP53* ^*R167H*^ expression on Oncopig STS transcriptional profiles combined with the ability to induce tumorigenesis in any cell type makes the Oncopig model a valuable resource for the development of further cell lines and *in vivo* sarcoma models.

## Materials and Methods

### Sample Collection

Oncopig (*Sus scrofa*) primary fibroblasts, AdGFP treated, and AdCre treated cell cultures from a previously published study were the source of RNA for this experiment^12^. All animal work was conducted according to relevant national and international guidelines, and approved by The University of Illinois Institutional Animal Care and Use Committee (IACUC; Protocol number 11221). Untreated (control), AdGFP, and AdCre cell lines were maintained separately for an average of 8, 16, and 31 passages after treatment, respectively (Supplementary Table S7). In addition, one AdCre cell line (63-3) was maintained in culture for 99 passages to assess the temporal stability of the transformed line.

### Immunocytochemistry

Formalin-fixed, paraffin-embedded primary fibroblast cell lines were sent to the Veterinary Diagnostic Laboratory (University of Illinois, Urbana, IL, USA) for vimentin and cytokeratin immunostaining to determine cell origins.

### RNA Isolation

Total RNA was extracted from each cell line using the AllPrep DNA/RNA Mini Kit (Qiagen, Valencia, CA, USA) following the manufacturer’s protocol. A NanoDrop spectrophotometer was used to determine RNA concentrations and RNA integrity and the presence of genomic DNA was determined using an Agilent 2100 Bioanalyzer using an RNA Nano bioanalyzer chip by the Carver High-Throughput DNA Sequencing and Genotyping Unit (HTS lab, University of Illinois, Urbana, IL, USA). RNA samples were utilized for sequencing if their RNA integrity number was greater than 7.

### RNA-seq Library Preparation

TruSeq Stranded RNA-seq libraries (TruSeq Stranded RNA Sample Preparation Kit, Illumina, San Diego, CA, USA) were produced from high-quality RNA (1 μg) by the HTS lab (University of Illinois, Urbana, IL, USA) following standard protocols. Qubit (Life Technologies, Carlsbad, CA, USA) was used to quantify libraries, and an Agilent bioanalyzer DNA7500 DNA chip (Agilent Technologies, Wilmington, DE, USA) was used to determine the average library size, which was diluted to 10 nM. Finally, library dilutions were quantitated using an ABI 1900 to ensure high accuracy quantification for consistent pooling and cluster maximization on the Illumina flowcell.

### Illumina Sequencing

RNA-seq was performed on an Illumina HiSeq2500. The libraries were paired-end sequenced to a total read length of 100 bp.

### RNA-Seq Data Analysis

An average of 34.7 million raw stranded paired-end reads were produced for each sample, ranging from 29.9 to 39.9 million (Supplementary Table S7). Trim Galore v.0.3.3 (http://www.bioinformatics.babraham.ac.uk/projects/trim_galore/) was used to trim raw reads for adapter contamination, A-tails, minimum quality score (20), and minimum length (20 bp) by setting the–stringency option to 6. A minimum length of 35 bp was used to retain unpaired reads. Trimmed reads were aligned to the swine reference genome (Sscrofa10.2)^58^ using Tophat v.2.2.10^59^ using the –M, –G, and fr-firststrand options, setting the –g option to 20, the –read-realign-edit-dist option to 0, and the –mate-std-dev option to 450. Previously aligned RNA-seq bam files for control Oncopig skeletal muscle (3) and leiomyosarcoma tumors (4) produced via injection of AdCre into the skeletal muscle were downloaded from the ArrayExpress database (www.ebi.ac.uk/arrayexpress) under accession number E-MTAB-3382. The bam files were aligned in the same manner as described above^12^, and the number of reads/sample is listed in Supplementary Table S7. Aligned bam files from *in vitro* and *in vivo* experiments were assessed for differential gene expression using cufflinks v.2.2.1^60^. Transcripts were assembled for each sample using the fr-firststrand option and merged together with the reference transcripts using Cuffmerge. Gene expression levels were pre-computed using Cuffquant with the –u and fr-firststrand options. Normalized gene expression levels were calculated using Cuffnorm using the fr-firststrand option and with the–library-norm-method set to geometric. Finally, differential expression was assessed using Cuffdiff including the –u and fr-firststrand options. Genes were considered differentially expressed with a q-value < 0.05. Samtools mpileup^61^ was used to determine WT:mutant *TP53* and *KRAS* expression ratios based on the reads overlapping the mutation site. The Shapiro-Wilk normality test was used to test normality, and equality of variance between groups was determined using an F-test. A two-way Student’s t-test was used to identify significant differences between groups for normally distributed data, while the Wilcoxon signed-rank test was used for non-normally distributed data. Correlations were performed using a Spearman’s rank correlation.

#### GO term and KEGG pathways analysis

GO term and KEGG pathway enrichment analysis was performed using DAVID v6.7^62^. DEGs were uploaded in the online database and all genes were used as a background. The Benjamini-Hochberg method was used to correct P values for multiple testing, and GO terms and pathways with a q value < 0.05 reported as enriched.

#### Master Regulators

Master regulators responsible for genome-wide increases and decreases in gene expression identified using the cytoscape plugin iRegulon v1.3^63^, which utilizes transcription factor binding motif information from 10 vertebrate genomes. DEGs displaying elevated and reduced expression were analyzed separately to identify master regulators responsible for widespread increases and decreases in expression, respectively. Transcription factor binding motifs with a normalized enrichment score (NES) > 4 were considered enriched.

## Electronic supplementary material

Supplementary Tables

Supplementary Figures
