# Supplementary material for: Oncopig Soft-Tissue Sarcomas Recapitulate Key Transcriptional Features of Human Sarcomas
Source: Sci Rep. 2017 Jun 1;7:2624. doi: 10.1038/s41598-017-02912-9 (PMC5453942; doi:10.1038/s41598-017-02912-9)
Supplement: Supplementary file 2 — Supplementary Figures [file 41598_2017_2912_MOESM2_ESM.pdf]

Supplementary Figures

Oncopig Soft-Tissue Sarcomas Recapitulate Key Transcriptional Features of Human Sarcomas

Kyle M. Schachtschneider, Yingkai Liu, Suvi Mäkeläinen, Ole Madsen, Laurie A. Rund, Martien A.M. Groenen, Lawrence B. Schook

Supplementary Fig. S1. Oncopig fibroblasts are mesenchymal in origin.

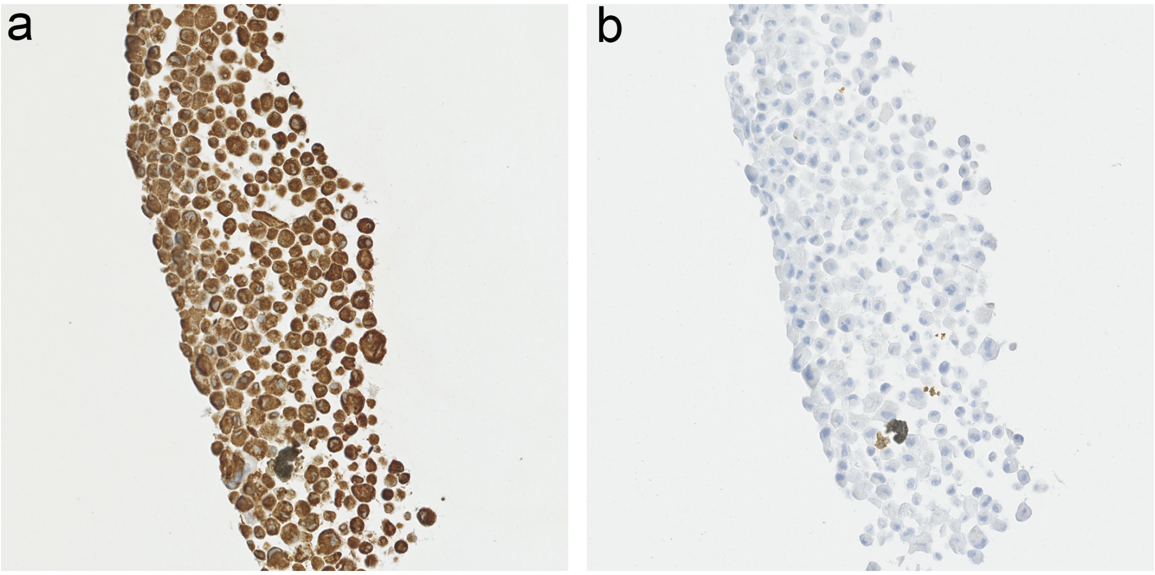

Oncopig primary fibroblasts stained a) positive for vimentin and b) negative for cytokeratin.

Supplementary Fig. S2. Temporal stability of Oncopig STS cell line expression profiles.

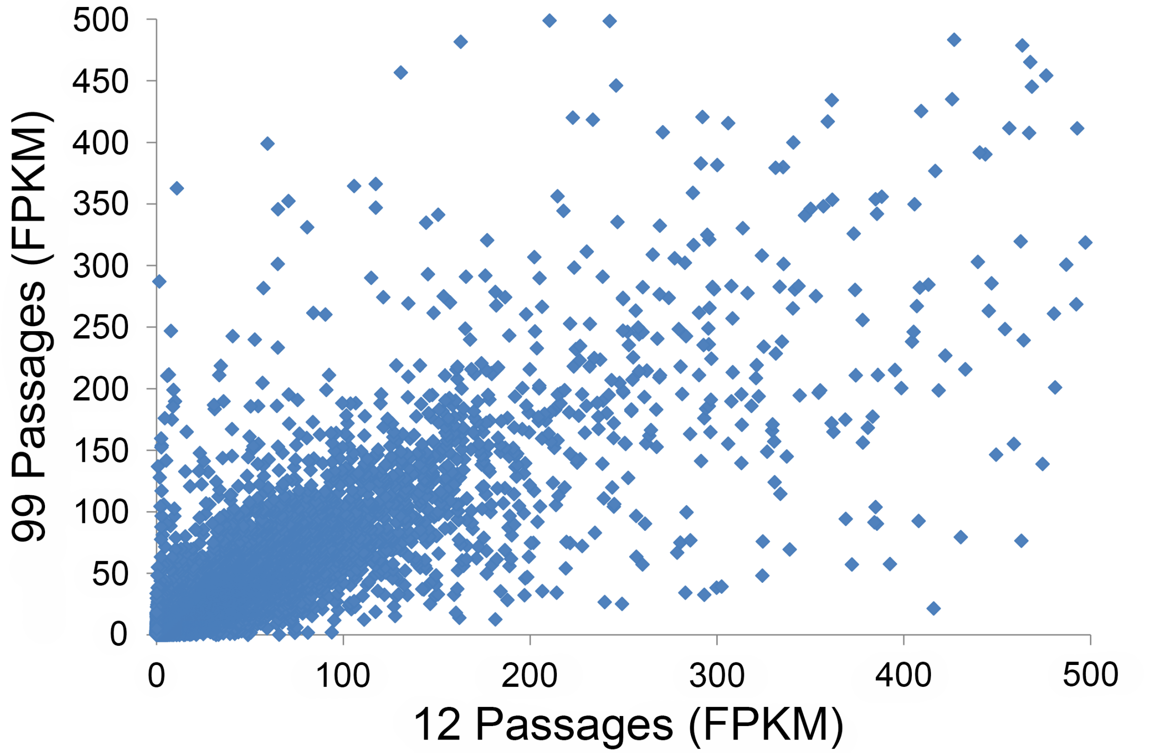

Expression profiles of one Oncopig STS cell line (63-3) maintained in culture for 12 and 99 passages were highly correlated (Spearman's Rho 0.92, $p < 1x10^{-15}$). Expression values are presented as fragments per kilobase of transcript per million fragments mapped (FPKM).
